# Dynamic tracking of human umbilical cord mesenchymal stem cells (hUC-MSCs) following intravenous administration in mice model

**DOI:** 10.1016/j.reth.2024.01.003

**Published:** 2024-01-24

**Authors:** Sze-Piaw Chin, Marini Marzuki, Lihui Tai, Nurul Ashikin Mohamed Shahrehan, Christine Ricky, Audrey Fanty, Annas Salleh, Chui Thean Low, Kong-Yong Then, Susan Ling Ling Hoe, Soon Keng Cheong

**Affiliations:** a Cytopeutics Sdn Bhd, Cyberjaya, Selangor, Malaysia; b Molecular Pathology Unit, Cancer Research Centre, Institute for Medical Research, NIH, Setia Alam, Selangor, Malaysia; c Department of Veterinary Laboratory Diagnosis, Faculty of Veterinary Medicine, Universiti Putra Malaysia, Serdang, Selangor, Malaysia; d M. Kandiah Faculty of Medicine and Health Sciences, Universiti Tunku Abdul Rahman, Sungai Long, Selangor, Malaysia

**Keywords:** Human umbilical cord mesenchymal stem cell, Biodistribution, GFP-Luc2

## Abstract

**Introduction:**

In the past decades, human umbilical cord-derived mesenchymal stem cells (hUC-MSCs) have sparked interest in cellular therapy due to their immunomodulatory properties. Nevertheless, the fate of hUC-MSCs in the body remains poorly understood. This study aimed to investigate the biodistribution, homing and clearance of systemically administered hUC-MSCs in healthy BALB/c mice model.

**Methods:**

hUC-MSCs were labelled with GFP-Luc2 protein, followed by characterisation with flow cytometry. Upon intravenous infusion of transduced hUC-MSCs into the healthy BALB/c mice, the cells were dynamically monitored through the bioluminescent imaging (BLI) approach.

**Results:**

Transduction of hUC-MSCs with GFP-Luc2 not only preserved the characteristics of MSCs, but also allowed live monitoring of transduced cells in the mice model. Upon systemic administration, BLI showed that transduced hUC-MSCs first localised predominantly in the lungs of healthy BALB/c mice and mainly remained in the lungs for up to 3 days before eventually cleared from the body. At terminal sacrifice, plasma chemistry biomarkers remained unchanged except for C-peptide levels, which were significantly reduced in the hUC-MSCs group. Histopathological findings further revealed that hUC-MSCs infusion did not cause any adverse effects and toxicity to lung, liver and heart tissues.

**Conclusions:**

Collectively, systemically administrated hUC-MSCs was safe and demonstrated dynamic homing capacity before eventually disappearing from the body.

## Highlights

• GFP-Luc2-lentiviral transduction did not alter hUC-MSCs basic characteristics.
• GFP-Luc2-transduced hUC-MSCs allowed dynamic monitoring in mice model.
• HUC-MSCs mainly localised in the lungs up to 3 days before cleared from the body.
• Intravenously infused hUC-MSCs did not cause any adverse events or toxicity in mice.

## 1 Introduction

Mesenchymal stem cells (MSCs) have emerged as one of the most promising and intensely pursued cellular therapies over the past decade due to their profound immunomodulatory and regenerative properties [1,2]. Among various adult and perinatal sources, it has been reported that the umbilical cord of a new-born child generally contains very rich and energetic MSCs [[3], [4], [5], [6]]. Umbilical cord is a postpartum medical waste sample with a minimal ethical issue, and the collection and isolation procedure of human umbilical cord-derived MSCs (hUC-MSCs) is painless and non-invasive [4]. Previous studies have demonstrated that hUC-MSCs have greater expansion capability, faster growth rate and a low expression of class I and II major histocompatibility complex in comparison to MSCs of other sources, indicating the added advantage for their subsequent downstream application in an allogeneic setting [[7], [8], [9], [10]]. Moreover, there is no tumorigenicity associated with UC-MSCs, confirming their safety for cell-based therapies [9,11,12].

Earlier, we isolated and expanded allogenic hUC-MSCs of young donors to study their safety and efficacy in the treatment of various inflammatory-related diseases. The safety and efficacy of our hUC-MSCs were demonstrated in *vivo* study [13], and in clinical studies involving healthy subjects [14], patients with diabetes [15] and knee articular cartilage defect [16], as well as in currently ongoing graft versus host disease (GvHD) clinical trial [17]. Specifically in the healthy volunteers, we have shown that hUC-MSCs treatment were safe and were able to reduce pro-inflammatory cytokines and to increase anti-inflammatory cytokines [14]. In these studies, hUC-MSCs were administered systemically by intravenous infusion (i.v.) as it was less invasive.

Preclinical data on biodistribution of cell-based therapy products like MSCs are mandatory for approval from local and international regulatory authorities (EMA Directive 2001/83/EC, 2013), to demonstrate that MSCs do not cause unwanted homing in organs that will subsequently induce uncontrolled differentiation, leading to tumour development. Although the safety and efficacy of hUC-MSCs had been studied, the data with regard to the biodistribution and fate of hUC-MSCs inside a living host is still not well explored. Previous literature have reviewed several approaches to track administered MSCs *in vivo*. These cells can be labelled with intracellular dyes, radioactive tracer molecules or nanoparticles, and subsequently tracked by using quantitative PCR (qPCR) of human specific gene sequences, *in vivo* live cell imaging or by histology after labelling with human specific antigens [[18], [19], [20]]. Due to the limitation of the direct labelling method, reporter gene detection system have become more suitable and feasible. For instance, luciferase-green fluorescent protein (Luc-GFP) was used to track the dynamic distribution of human bone marrow MSCs (BM-MSCs) in Winstar rats or immunodeficient mice models using bioluminescent imaging (BLI). Both studies had reported that upon tail vein injection, BM-MSCs mainly reside in the lung region, and the signals steadily decreased over time until could not be detected within 3–5 days. Meanwhile, BLI signals were also detected in the back skin and kidney of the rats, and in the abdomens of the mice, and these signals eventually weakened and undetectable by day 14 [21,22].

Here, the study was designed by referring to the current literature of similar nature [23]. Sadeghi and team (2019) have reported that transduced placenta-derived decidua stromal cells (DSCs), a type of MSC-like cells, localised in the lungs of healthy BALB/c mice up to 4 days after infusion, and no signals were detected in liver or spleen during the evaluation period. No immediate toxicity or adverse events was reported in the mice [23]. We have chosen the transduction method of hUC-MSCs that enables promoter-activated surrogate luciferase activity to be detected with BLI. This is because BLI is relatively low-cost, highly sensitive, high-throughput, and most importantly allows for the non-invasive and non-radioactive monitoring of luciferase-transduced cells in living animals [21,24]. Besides, luciferase is a specific marker of live cells as the signal is dependent on the release of enzyme luciferase, which enables evaluation of how fast hUC-MSCs are cleared systemically [25].

This study aims to demonstrate the transducibility of hUC-MSCs by lentiviral vectors and to explore the biodistribution, homing, as well as clearance of hUC-MSCs in healthy BALB/c mice, following i.v. administration.

## 2 Materials and methods

### 2.1 Cell culture and media

Cytopeutics Sdn. Bhd. owns good manufacturing practice (GMP)-synthesised human umbilical cord-derived mesenchymal stem cells (hUC-MSCs) and acts as a stem cell provider for their use as the investigational product for in this study. Briefly, human umbilical cord samples were collected after delivery of full-term, healthy babies with written consent from both parents. All three generations (newborn, parents and grandparents) were screened for genetic mutations, infections, cancers, and other inherited diseases before the samples were transferred to the laboratory for processing. All cell processing was done in a certified GMP laboratory in accordance with the Malaysian Guidelines for Stem Cell Research and Therapy, and the National Committee on Ethics in Cell Research and Therapy (NCERT). Isolation and culturing had been established and reported previously [14]. The hUC-MSCs were cultured with GMP-grade Dulbecco's Modified Eagle Medium (DMEM) low glucose (ThermoFisher Scientific, USA) media supplemented with 5 % Stemulate™ pooled human platelet lysate (Cook General BioTechnology, USA) in 37 °C, 5 % CO_2_ incubators. The cells provided for this study were tested negative for murine pathogens, mycoplasma, endotoxins and other infectious agents.

### 2.2 Labelling of hUC-MSCs with green fluorescent protein-luciferase protein (GFP-Luc2)

Lentiviral transduction was performed according to previous protocols with some modifications [26]. hUC-MSCs were transduced with a *gfp-luc2* lentiviral construct.

#### 2.2.1 Transfection of lentivirus with *gfp-luc2* genes, and concentration and storage of transfected lentivirus

The HEK293T (ATCC, USA) cells were seeded (3.0–3.5 × 10^6^ cells per 10-cm dish) one day before transfection. On the transfection day, a plasmid master mix was prepared (Table 1). Old media were removed and 8 ml of fresh DMEM with 10 % FBS media without Pen/Strep were added to each of HEK293T dish. About 2 ml plasmid master mix were added into each dish. Cells were incubated for 6 h at 37 °C with 5 % CO_2_. After 6 h of incubation, media were discarded and 10 ml of fresh media with Pen/Strep (ThermoFisher Scientific, USA) were added into the dish. Cells were incubated overnight at 37 °C with 5 % CO_2_. Lentiviral supernatant was collected every day (at 24, 48 and 72 h post-transfection) and the supernatant was kept in a sterile tube and stored at 4 °C. After supernatant collection, 10 ml of fresh media with Pen/Strep were added into the same plate. The cells were incubated again at 37 °C with 5 % CO_2_. After 72 h post-seeding, all the collected lentiviral supernatant was pooled and filtered using PVDF Millex-HV filter, 0.45 μm (Merck, Millipore, USA) and the filtered supernatant was transferred to a sterile tube. One volume (10 ml) of Lenti-X Concentrator (ThermoFisher Scientific, USA) was added with 3 volumes (30 ml) of lentiviral supernatant (at the ratio 1:3). The mixture was mixed gently by inversion and incubated at 4 °C for overnight.

**Table 1:** List of plasmid master mix (total volume for 3 dishes).

Reagents	Tube A	Tube B
Opti-MEM (ThermoFisher Scientific, USA)	3000 μl	3000 μl
Lipofectamine 2000 reagent (ThermoFisher Scientific, USA)	–	120 μl
pMDL (5 μg)	5 μg x 3	–
RSV-Rev (2.5 μg)	2.5 μg x 3	–
VSV (3 μg)	3 μg x 3	–
Ful-2TG (10 μg)	10 μg x 3	–
Mix Tube A and Tube B (1:1) - Incubate for 10–20 min at RT	3000 μl	3120 μl
	Total = ∼6 ml	

After overnight, lentiviral-Lenti-X mixture was centrifuged at 1500×*g* for 45 min at 4 °C. The supernatant was removed and the lentiviral pellet (1/10 of original volume) was resuspended using 3 ml of complete DMEM media. Lentiviral suspension was aliquoted in single-use volumes (500 ul/tube) and stored at −80 °C freezer. The freezing and thawing activities of the stored lentiviral suspension should be minimized; there were 15–20 % expected reduction in virus titre for every freeze–thaw cycle. Aliquoting was performed on ice.

#### 2.2.2 Transduction of lentivirus into target cells

A day before transduction, 0.5 × 10^6^ hUC-MSCs per well of 6-well plate were seeded and incubated overnight. On the day of transduction, lentiviral supernatant vials were thawed on ice a few mins prior to usage. Five minutes before transduction, the vials were removed from ice and allowed to equilibrate to room temperature. Once thawed, the lentivirus was used for transduction as soon as possible to avoid degradation.

The hUC−MSCs culture medium was also first allowed to fully equilibrate to room temperature to prevent cold shock. Polybrene (Merck, Millipore, USA) at the final concentration 10 μg/mL was then added into the culture medium. Five hundred microliter culture media with polybrene and 500 ul of lentiviral supernatant were added to each well. The plate was centrifuged at 1200×*g* for 2 h at room temperature and later, the plate was incubated overnight at 37 °C with 5 % CO_2_. After overnight (24 h post-transduction), the medium containing virus was removed from the wells and replaced with 2 ml of fresh culture medium (without polybrene). GFP signal was clearly visible after 2–3 days post-transduction in GFP-Luc2-hUC-MSCs (hereinafter referred to as transduced hUC-MSCs).

### 2.3 *In vitro* characterisation of transduced hUC-MSCs

The transduction efficiency of *gfp-luc2* lentiviral vector into hUC-MSCs was monitored by inverted fluorescent microscope (Olympus CKX41, Germany) at 24, 48 and 72 h post-transduction. Mean fluorescence intensity of the images was analysed using ImageJ 1.5i software (NIH, USA). The percentage of GFP-expressing (GFP-positive) transduced hUC-MSCs was determined by flow cytometry analysis (BD FACSAria™ SORP, USA) over a period of 14 days in culture.

Furthermore, these cells were tested for expression of the following surface markers: cluster of differentiation (CD)105, CD90, CD70 and CD44, and negative cocktails comprise of CD45, CD34, CD11b, CD19 and human leukocyte antigen-DR isotype (HLA-DR) (BD Stemflow, Cat No. 562245) according to the manufacturer's instructions by using flow cytometry (BD FACSLyric™, USA). Fluorescence intensity of 10^4^ cells was recorded and analysed using BD FACSDiva™ Software and BD FACSuite™ Clinical Software.

### 2.4 *In vitro* bioluminescent imaging (BLI)

To determine the detection threshold of transduced hUC-MSCs in IVIS® Spectrum Imaging System (PerkinElmer, USA), different numbers of cells (1, 5 and 10 × 10^3^ cells) were seeded into a black multi-label plate reader (PerkinElmer, USA) and incubated with D-luciferin. D-luciferin solution was added to activate the luciferase reporter and the metabolic activity. Bioluminescent signals detected by IVIS® were used to generate kinetic curves of transduced hUC-MSCs.

### 2.5 Animal model

The study protocol was approved by the Animal Care and Use Committee (ACUC), Ministry of Health Malaysia (MOH) (Approval Number: ACUC/KKM/02(09/2021) (9)). A total of 12 BALB/c mice (8- to 12-week-old, 25–30 g; Nomura Siam International Co. Ltd., Thailand) were used in this study. All mice were kept in sterilised polycarbonate cages in a controlled room at 19–24 °C with a 12-h light/dark cycle, relative humidity of 40–70 %, and free access to food and water. The mice were acclimatised in the experimental room for 7 days prior to the initiation of dosing, and clinical signs were observed daily throughout the acclimatization period. Mice were randomly allocated into two groups: (i) saline - mice received normal saline injection (n = 6), and (ii) transduced hUC-MSCs - mice received single i.v. injection of transduced cells at the dose of 0.46–0.50 × 10^6^ cells (equivalent to ∼18.5 × 10^6^ cells/kg body weight, BW in mice) (n = 6).

At 72 h post-transduction, transduced hUC-MSCs were trypsinised and collected using centrifugation (600×*g* for 5 min at room temperature). The cells were resuspended in normal saline (vehicle). The suspension was administered through i.v. slow bolus injection in lateral tail vein using 1 mL syringe with 25-gauge needle (25 G). The injection volume was maintained as 0.2 ml/mouse and the injection was completed within 30 min after cell preparation. Saline-treated mice received single injection of saline only at 0.2 ml/mouse.

### 2.6 *In vivo* characterisation of transduced hUC-MSCs

#### 2.6.1 *In vivo* bioluminescent imaging (BLI)

For *in vivo* BLI, imaging was performed on each mouse for up to 7 days or until the bioluminescence signal was not detectable. Prior to each imaging timepoint, the mice were injected with luciferin (intraperitoneal, i.p.) and anaesthetised with isoflurane supplied with oxygen. All the mice were imaged at dorsal and ventral positions. Bioluminescence signals of whole live mice and individual organs were quantified by regions of interest (ROIs) and recordings of total flux (photon/second). The relative signal intensity from each organ was calculated as percentage of the signal intensity from the whole body. Since i.v. injection through tail vein can lead to small fraction of cells trapped in or around the injection site, causing strong signal intensities, the total flux of the tail area was subtracted from the total flux of whole body.

To determine the optimum signal pick-up time of transduced hUC-MSCs in mice, images were captured every 2 min until the signal peaked and a signal plateau was reached. The bioluminescence signals were then used to generate an *in vivo* kinetic curve for luciferase expression *in vivo*. Quantification of the bioluminescence signals in *vivo* experiments were recorded.

#### 2.6.2 Clinical and surgical pathology

All mice were observed individually for clinical signs once daily. Clinical parameters included body weight, physical examination (i.e. body temperature, fur aspect, posture, urination, diarrhoea, eye and nose discharges), behaviour (i.e. active, depress) and food consumption. Clinical observations during acclimatisation phase are not reported.

#### 2.6.3 Blood collection, plasma isolation and clinical chemistry parameters

Terminal blood collection was carried out in all animals at the end of the observation period for biomarker analysis. Blood was collected via submandibular vein of animals into K2EDTA-coated tubes. Plasma was separated by centrifugation at 4 °C at 2000 rpm for 15 min. Plasma samples were stored at −80 °C for subsequent biomarker analysis.

The plasma chemistry biomarkers such as aspartate aminotransferase (AST), alanine aminotransferase (ALT), amylase, creatinine, C-peptide, N-terminal pro-brain natriuretic peptide (NT-proBNP), and cardiac troponin I (cTnI) were analysed for the evaluation of general health of the animals.

#### 2.6.4 Hematoxylin-eosin (H&E) staining

All animals were euthanised by cervical dislocation at the end of the observation period (24 h and day 7). Excised organs were preserved in 10 % buffered formalin for 24 h, routinely processed, and embedded in paraffin. The tissues were sectioned into 5-μm-thick sections, stained with H&E, and subjected to histopathological examination in a blinded manner.

### 2.7 Statistical analysis

Statistical analysis was performed using Graph Pad Prism version 9 for Windows (Graph Pad Software, San Diego, CA). Statistical significance for BLI intensity and plasma chemistry parameters were determined by one-way ANOVA with Tukey multiple comparison test and unpaired t-tests, respectively, to compare the means between two groups. The *p*-value of less than 0.05 (*p* < 0.05) was considered statistically significant. The data were presented as mean ± standard error mean (SEM) from independent experiments, except for flow cytometry data which were presented as mean ± standard deviation (SD).

## 3 Results

### 3.1 *In vitro* characterisation of transduced hUC-MSCs

The cellular morphology and efficiency of transduction in transduced hUC-MSCs were evaluated under inverted microscopy. The transduced cells showed similar morphology to the parental hUC-MSCs, which indicated that the transduction process did not alter cellular morphology.

**Fig. 1:**
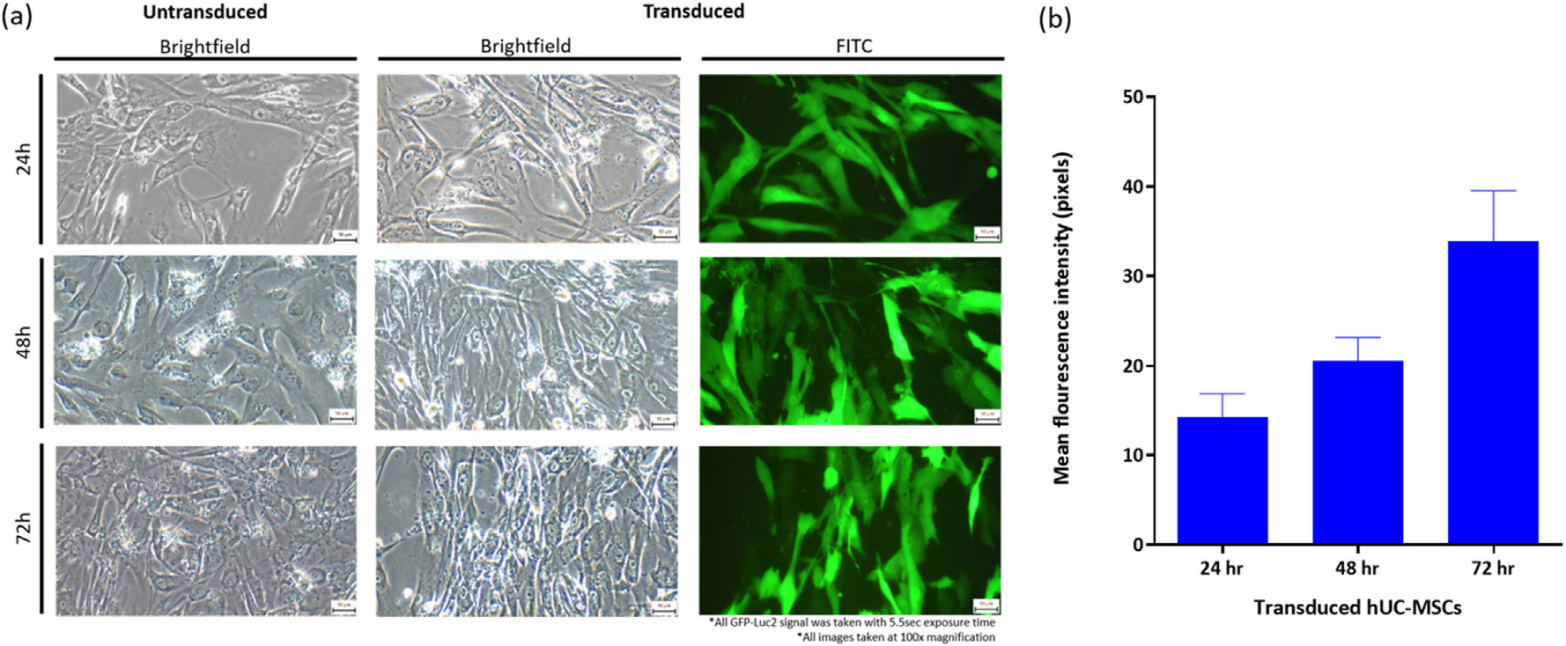
Morphology and GFP-Luc2 expression in parental hUC-MSCs and transduced hUC-MSCs at 24, 48 and 72 h post-transduction. (a) Transduction of GFP-Luc2 proteins in hUC-MSCs did not cause morphological changes. All images taken at 100x magnification. The scale bars represent 50 μm. The GFP signals were taken with 5.5 s exposure time. (b) Mean fluorescence intensities of transduced hUC-MSCs at 24, 48 and 72 h post-transduction (n = 2).

Transduction efficiency and growth of the transduced cells were monitored under fluorescence microscope at three different time points (24, 48 and 72 h) post-transduction. At 24 h post-transduction, low GFP signals were detected. After 48 h post-transduction, the number of cells expressing GFP had increased, and reached the highest signal intensity at 72 h (Fig. 1a). Calculated mean fluorescence intensity of the cell images taken at those time points also corroborated with the microscopy observations (Fig. 1b). Therefore, the optimal transduction time for GFP-Luc2 transduction into parental hUC-MSCs was chosen at 72 h for subsequent experiments.

**Fig. 2:**
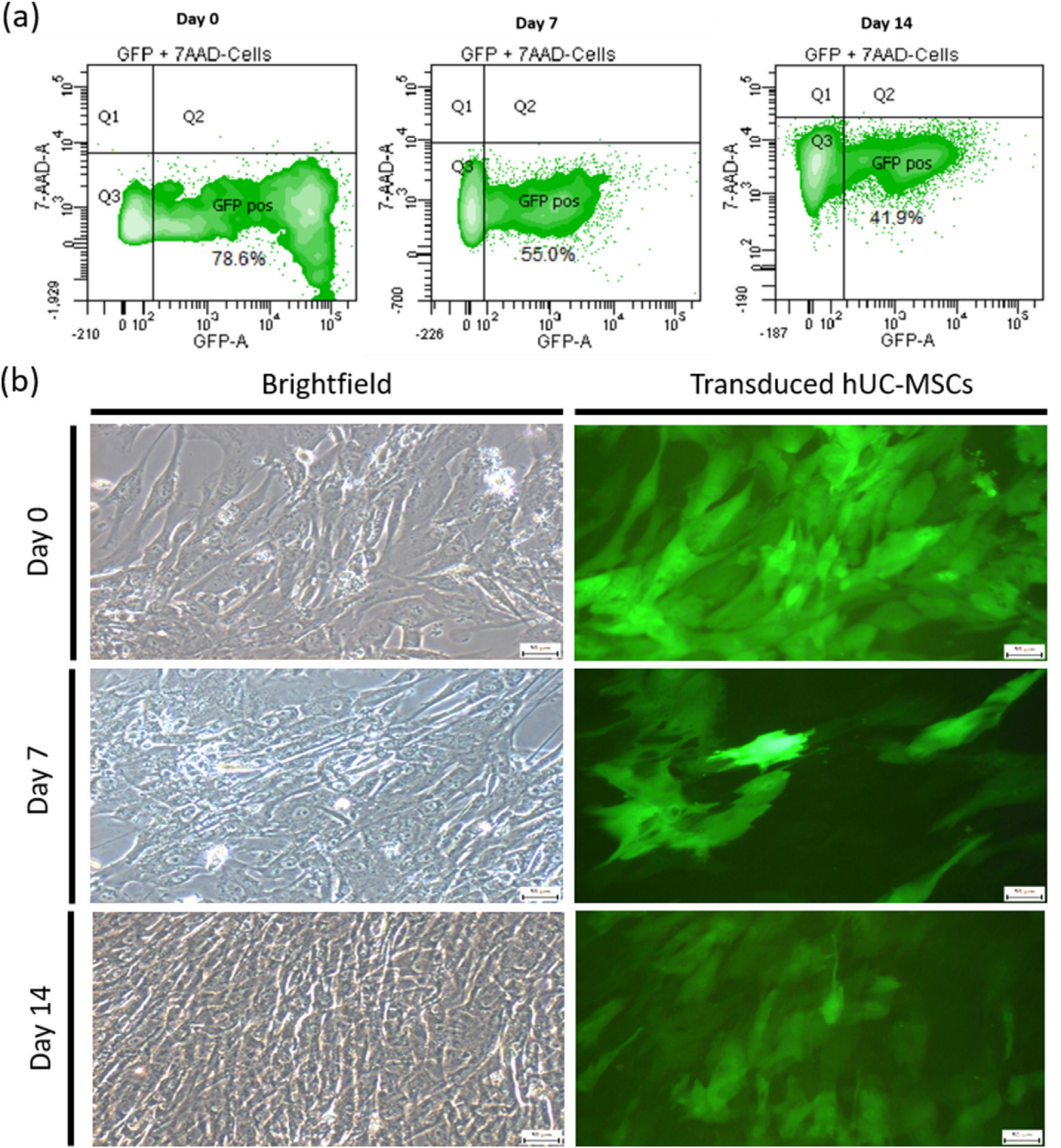
Time course analysis of GFP expression in transduced hUC-MSCs. (a) Representative histogram showed the percentage of GFP-positive cells at day 0, 7 and 14. (b) Representative microscopic images of transduced hUC-MSCs (GFP-Luc2) at day 0, 7 and 14 in culture. All images taken at 100x magnification. The scale bars represent 50 μm. The GFP signals were taken with 5.5 s exposure time.

The stability of GFP expression in transduced hUC-MSCs was further assessed by examining the GFP expression level (percentage of GFP-expressing cells) for up to 14 days in culture (Fig. 2). The GFP expression was evaluated by flow cytometer at three different time points (day 0, 7 and 14) in culture. Following 72 h post-transduction (day 0), 73.1 ± 5.5 % of the total cell population was found to be GFP-positive. The percentage of GFP-expressing cells decreased to 46 ± 9.05 % at day 7, prior to stabilising at 40 ± 1.4 % for additional 7 days in culture (day 14) (representative dot plots in Fig. 2a). Fluorescence microscopy images complemented with the flow cytometry data to show a similar decrease in the number of GFP-expressing cells after 7 days in culture (Fig. 2b).

#### 3.1.1 Expression of MSCs markers in transduced hUC-MSCs

**Fig. 3:**
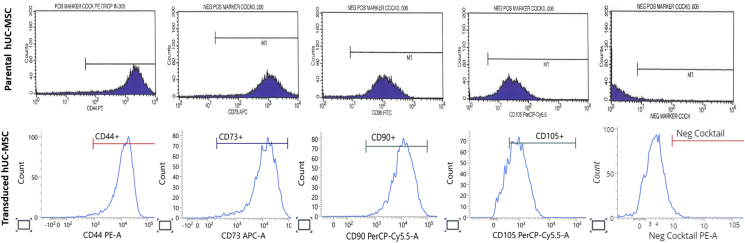
Immunophenotyping of MSCs surface markers in hUC-MSC. Representative histograms of parental hUC-MSCs and transduced hUC-MSCs for the MSCs specific surface markers CD44, CD73, CD90 and CD105, and negative cocktails consisting of CD45, CD34, CD11b, CD19 and HLA-DR. The gating for cells was based on its respective unstained control.

To further evaluate whether transduction had affected the stem cell phenotype, transduced hUC-MSCs were profiled for MSC-specific surface markers such as CD73, CD90, CD105 and CD44, and negative markers including CD45, CD34, CD11b, CD19 and HLA-DR by flow cytometry (Fig. 3). Table 2 shows that the transduced hUC-MSCs had consistent marker expression levels with parental cells: strongly positive for CD73 (99.3 ± 0.22 %), CD90 (99.6 %) CD105 (77.5 ± 1.03 %), and CD44 (98.2 ± 0.28 %), with a relatively low expression of negative markers (3.6 ± 0.30 %), thus confirming that the stemness of the cells were unaffected by the GFP-Luc2-lentiviral transduction.

**Table 2:** Immunophenotyping of MSCs surface markers in hUC-MSCs.

MSCs CD marker	Percentage of cells positive for MSCs surface markers (%)	
	Parental hUC-MSCs^a^	Transduced hUC-MSCs
CD73	98.2	99.3 ± 0.22^b^
CD90	97.5	99.6^c^
CD105	95.6	77.5 ± 1.03^b^
CD44	96.4	98.2 ± 0.28^b^
Negative markers	1.8	3.6 ± 0.30^b^

Negative markers consist of CD45, CD34, CD11b, CD19 and HLA-DR.

a Characterisation prior to GFP-Luc2 transduction.

b Data are represented as mean ± SD from two independent experiments.

c Data from one experiment.

### 3.2 *In vitro* BLI of transduced hUC-MSCs

**Fig. 4:**
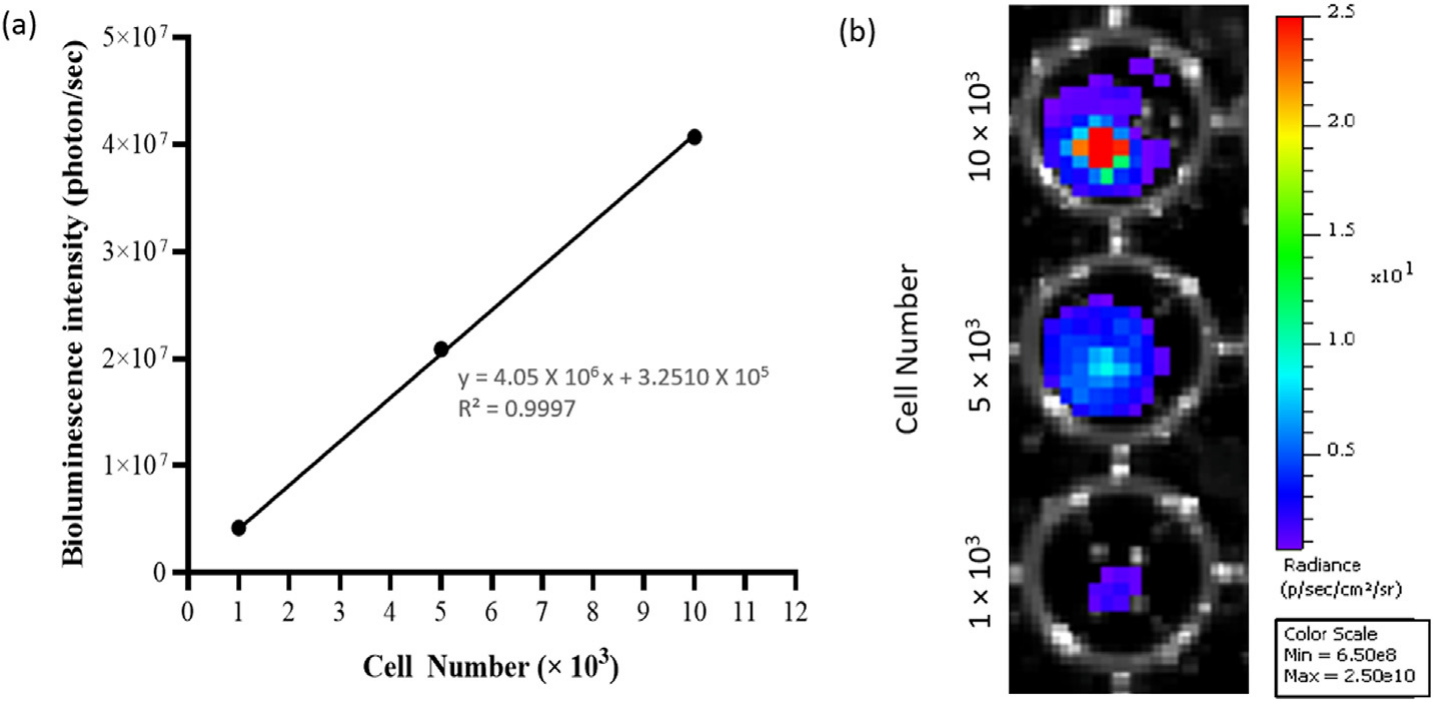
Linear relationship analysis between the cell number and bioluminescence intensity. (a) Quantitative analysis revealed a strong linear relationship between cell numbers (1, 5 and 10 × 10^3^ cells) and the bioluminescence signal (R^2^ = 0.9997). (b) Representative images of at least 2 to 3 independent experiments.

Prior to conducting BLI in the mice, the detection threshold and robustness of IVIS® Spectrum Imaging System was first determined using cultured transduced hUC-MSCs. There was a strong correlation between bioluminescence intensity signals and cell numbers (R^2^ = 0.9997; Fig. 4). Additionally, this demonstrated the successful integration and activation of the GFP-Luc2 exogenous proteins in hUC-MSCs, which provides an ideal tool for the tracking and quantification of transplanted transduced hUC-MSCs in animal model.

### 3.3 *In vivo* characterisation of transduced hUC-MSCs

#### 3.3.1 *In vivo* BLI to monitor the biodistribution of transduced hUC-MSCs

**Fig. 5:**
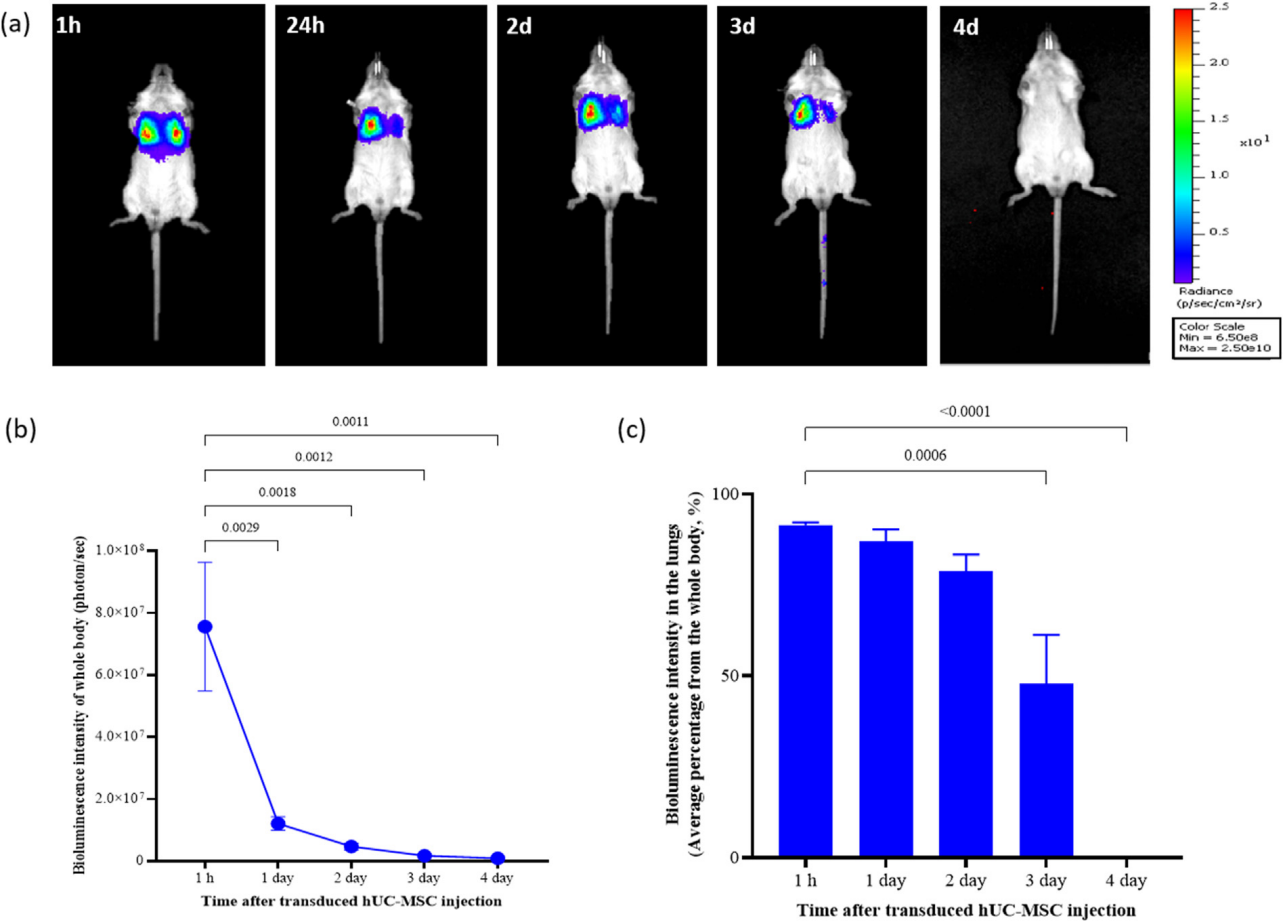
Representative images depicting biodistribution pattern of single i.v. administered transduced hUC-MSCs (18.5 × 10^6^ cells/kg BW) in healthy BALB/c mice at designated time points (1 h, 24 h, 2, 3, 4 days after cell administration). (a) Transduced hUC-MSCs migrated and localised to lungs as early as 1 h post-administration. The signal remained in lungs for up to 3 days before being cleared from the body. (b) Bioluminescence intensity quantification of the whole body of mice at designated time points. (c) Average percentage of signal intensity in lungs at designated time points. Data are presented as mean ± SEM (n = 5).

To investigate the trafficking, homing, engraftment, as well as clearance of transduced hUC-MSCs after i.v. administration in healthy BALB/c mice, the distribution of cells to various organs was monitored by BLI at various time intervals until the signal intensity disappeared (Fig. 5).

At day 0, a single, slow i.v. bolus injection of transduced hUC-MSCs at the dose of 0.46–0.50 × 10^6^ cells (which is equivalent to dose of ∼18.5 × 10^6^ cells/kg BW in mice) were administered through tail vein of healthy mice. Immediately, IVIS imaging system detected an intense bioluminescence signal at the first hour after infusion, with the majority of transduced hUC-MSCs in the two lung lobes (91.4 ± 0.73 %; ventral view) (Fig. 5a). During the first 24 h, the whole-body bioluminescence intensity reduced significantly, but the signals were still concentrated in the lungs. The signals remained detectable in the lungs up to 3 days, with a reduction of the intensity to 47.8 ± 13.48 %, and eventually it could not be detected at day 4 post-administration. The complete disappearance of the signal suggests the clearance of the transduced cells from the body. Interestingly, no bioluminescence signal was detected in other main organs including liver, kidney or spleen at any evaluated time point.

#### 3.3.2 Physical and behavioural assessment in healthy BALB/c mice

**Fig. 6:**
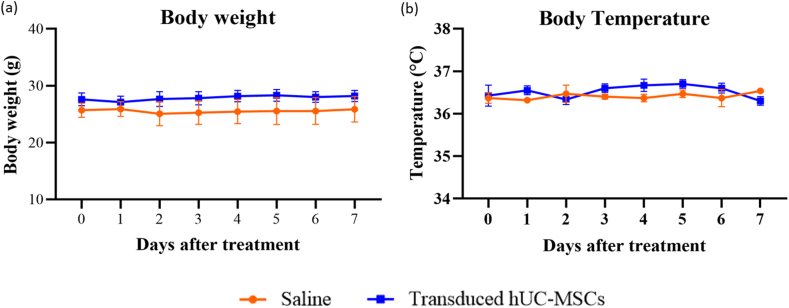
Time course of (a) body weight and (b) body temperature changes in mice from before i.v. administration (day 0) until the end of the study (day 7). Data are presented as mean ± SEM (n = 6 for Day 0–1; n = 3 for day 2–7).

Generally, the physical and behavioural assessment of mice did not present any significant difference throughout 7 days of the study period. None of them showed any immediate adverse effects including restless, breathing problem (dyspnoea) or any changes in grooming and activity following intravenous injection of transduced hUC-MSCs. There was no sign of morbidity nor mortality in both groups. There was no treatment-related change in body weight (Fig. 6a) and body temperature (Fig. 6b) in all animals.

#### 3.3.3 Plasma chemistry in healthy BALB/c mice

Analyses of plasma AST, ALT, creatinine, amylase, cTnI and NT-proBNP did not reveal statistically significant difference between saline and transduced hUC-MSCs groups at day 7 post-administration. However, C-peptide decreased significantly (*p* < 0.0001) in transduced hUC-MSCs group when compared to saline group (Table 3).

**Table 3:** Selected plasma chemistry parameters of mice on day 7 following single i.v. administration of transduced hUC-MSCs.

Parameters	Saline	Transduced hUC-MSCs
AST (U/L)	273.5 ± 113.8	60.6 ± 4.8
ALT (U/L)	119.7 ± 59.6	33.0 ± 1.16
Amylase (U/L)	2578.0 ± 242.3	2381.0 ± 132.7
Creatinine (μmol/L)	36.3 ± 0.89	74. 7 ± 22.56
C-peptide (ng/mL)	4.4 ± 0.08	1.9 ± 0.12∗
Cardiac troponin I (pg/ml)	76.0 ± 12.6	49.4 ± 3.0
NT-proBNP (pg/mL)	207.6 ± 78.1	87.5 ± 8.0

Data are presented as mean ± SEM (n = 3).*∗p < 0.0001 vs. saline-treated mice*.

### 3.4 Histopathology evaluation of lung, liver and heart following transduced hUC-MSCs administration

As reported above, transduced hUC-MSCs initially migrated and localised to lungs of mice as early as 1 h post-administration and remained in the lung for up to 3 days post-administration. Histopathology assessments showed mild pulmonary haemorrhage and mild infiltration of inflammatory cells in the alveolar spaces, as well as aggregation of mesenchymal cell in the lumen of a blood vessel in the lungs. The similar observation was also detected in the vehicle group. At the same time, the air sacs and alveolar regions appeared normal in both saline and transduced hUC-MSCs groups.

Histopathology evaluation of the liver at 24 h and day 7 post-treatment did not show any significant lesion related to the cell treatment. Both groups showed normal hepatic architecture without any observable lesion. Mild-to-moderate diffuse glycogenesis and moderate hepatocyte vacuolization were observed in all liver tissues. However, none of these observations were related to the transduced hUC-MSCs treatment.

In the heart sections, both saline and transduced hUC-MSCs-treated mice showed normal histological structure of myocardium, which appeared as branched and striated cardiac muscle fibres, with central oval vesicular nuclei. Considering all this, single i.v administration of transduced hUC-MSCs at a dose of 18.5 × 10^6^ cells/kg BW did not induce any of the prominent histopathologic features at 24 h and day 7 post-treatment in lungs, liver and heart of BALB/c mice.

**Fig. 7:**
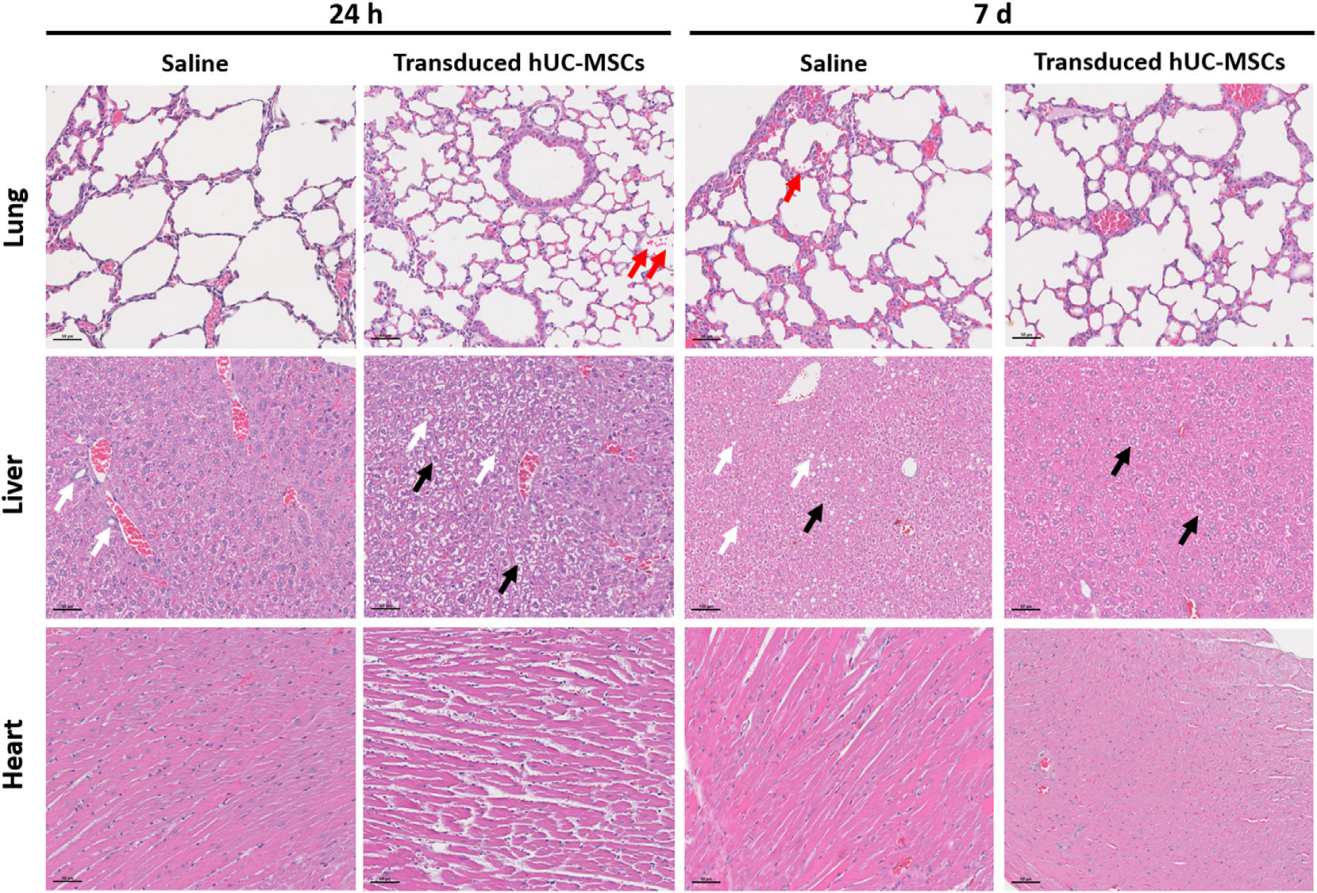
Representatives photomicrographs of the lung, liver and heart as stained by H&E. The lung, liver and heart tissues were found unaffected by the treatment of transduced hUC-MSCs, as evidenced by normal tissues architecture, without any observable lesion. Red arrows indicate pulmonary haemorrhage; black arrows indicate glycogenesis and white arrows indicate vacuolization. All images taken at 100x magnification. The scale bars represent 50 μm.

Representative photomicrographs of the lungs, liver, and heart at 24 h and day 7 were shown in Fig. 7.

## 4 Discussion

In the present work, a rapidly incorporated, highly sensitive and non-radioactive GFP-Luc2 protein cassette was used to monitor the systemic biodistribution of hUC-MSCs in healthy BALB/c mice after i.v. administration. The results showed that hUC-MSCs were transducible by lentivirus with relatively high uptake of the GFP-Luc2 exogenous proteins at 72 h. The GFP signal, encoded by *gfp* reporter gene, provided a fluorescent visualization of transduced hUC-MSCs *in vitro*. At day 7 in culture, the percentage of GFP positive cells was slightly reduced, which could be due to the outgrowth of non-transduced cells over transduced cells [27]. Nevertheless, the fluorescent signals of transduced hUC-MSCs remained to be detected up to day 14 in culture, signifying that the signals were adequately stable without bleaching. Whereas in *vivo* setting, the luciferase in the transduced hUC-MSCs, encoded by the *luc2* reporter gene, was able to react chemically with luciferin to emit luminescent signal that was proportional to the cell numbers, further supporting the feasibility of GFP-Luc2 in monitoring the distribution, localisation and clearance of hUC-MSCs in mice models.

In addition, lentiviral transduction does not disrupt the cellular morphology and surface markers protein expression of hUC-MSCs. Athiel et al. (2022) reported the absence of GFP lentiviral genomic DNA in their samples of lambs engrafted with allogenic UC-MSCs, thus providing a plausible explanation to our observations of no/minimal change to the morphology and protein expressions in hUC-MSCs [28]. The expression of CD44 in transduced hUC-MSCs was slightly higher compared to the parental cells which is in complement with the data reported by Lin et al. (2017) [29]. They showed that MSCs might obtain a higher level of CD44 expression after passaging in bone marrow-derived MSCs from mouse and human. However, the expression level of the CD105 marker was slightly dropped in the transduced hUC-MSCs, likely due to the shear stress received during the transduction and expansion process [30]. It was also reported previously that CD105 expression of various sources of MSCs, including bone marrow, adipose tissue and umbilical cord, were affected by different culture conditions, but their stemness and *in vitro* differentiation capability remained unchanged [31,32]. Furthermore, CD105 negative MSCs demonstrated stronger immunomodulatory capacity compared to CD105 positive MSCs [32]. Collectively, these data have suggested that the diminished expression of the CD105 marker of transduced hUC-MSCs does not affect the characteristics of the cells.

Upon i.v. infusion of transduced hUC-MSCs into the mice, the cells mainly travelled to and were distributed in the lungs before circulated systematically due to the pulmonary first-pass effect. Then, the cells remained in the lungs up to 3 days until no signal was detected at day 4 post-infusion. Aforementioned, the GFP fluorescent signals remained stable and were detected up to day 14 in culture, and luciferin/luciferase activity in cells has also previously been demonstrated to be functional for up to 8 days [26]. Hence the sudden loss of bioluminescent intensity in the mice at day 4 signifies that the transduced hUC-MSCs were being cleared from the body. The observation also suggested that transduced hUC-MSCs administration does not lead to engraftment and uncontrolled differentiation in the healthy living host. Nevertheless, it is possible that hUC-MSCs has distributed from the lungs to other organs, and the number of cells in these organs is too low and below the detection limit of BLI.

Our findings are consistent with a recent study that imaged the biodistribution of intravenously injected hUC-MSCs, labelled with tdTomato fluorescent reporter, in C57BL/6 mice. Whole-body BLI showed that the signal in the lungs was strong at 2 h and significantly weakened at 24 h, confirming the localisation of hUC-MSCs in the lungs [33]. More than 95 % of firefly luciferase-GFP transduced hUC-MSCs, from three different donors, were undetected within 3 days of infusion in immunocompromised and immune-competent mouse strains, with only a small portion detected until day 14 [34]. Besides, in another study that used the fluorescence tracing method, an intensified signal was reported in the lung at 30 min time point after injection of labelled hUC-MSCs, followed by a sudden decreased and complete disappearance of signal at day 7 [35]. Regardless of the different methods used to study the biodistribution of MSCs, our findings were consistent with those of previous works, which reported that i.v. administration of MSCs initially localised to the lungs of healthy mice, before migrating to other organs and eventually disappearing from the body [[20], [21], [22], [23]].

Furthermore, treating the mice intravenously with transduced hUC-MSCs at a dosage of 18.5 × 10^6^ cells/kg BW has no immediate or abnormal reaction and toxicity observed throughout the study. Even though the levels of several markers such as AST, ALT, amylase, cTnI and NT-proBNP had decreased, the reduction was not significant. Interestingly, the level of C-peptide at day 7-post transduced hUC-MSCs administration was significantly reduced, as compared to the control group receiving normal saline. C-peptide is an indicator of insulin secretion by functioning pancreatic beta cells, and high levels of C-peptide indicate possible resistance to insulin, beta cell neoplasm or kidney disease [36]. It was speculated that this decrease is due to the immunomodulation properties of hUC-MSCs indirectly regulating the production of C-peptide in mice. Nonetheless, the C-peptide level was comparable to those previously reported in C57BL/6 mice (approximately 2 ng/mL) receiving a normal chow diet [37]. Generally, the results suggested that transduced hUC-MSCs infusion does not affect the blood biochemistry profiles of liver, pancreas, kidney and cardiac, which was consistent with the literature [23].

Following, histopathology examination has unveiled minor pulmonary haemorrhage and cell aggregates in the lung vasculature of mice in both vehicle and treatment groups, which was also reported in previous study [23]. In fact, the study by Pichardo and team, which used deep tissue imaging with optical-tissue clearing, found that after i.v. administration, hUC-MSCs were retained in lung microvasculature, but not in the macrovasculature, and were accompanied by rapid infiltration of neutrophils [33]. Similar observations were reported in another study, which also identified that hUC-MSCs administration elevated neutrophil levels in the lungs, blood and spleen, and increased plasma neutrophils chemoattractants, thereby eliciting immune reaction [34]. Besides, minor glycogenesis was observed in the livers at 24 h and day 7 post-treatment. This is because prior to euthanasia, the mice did not experience fasting, and the diet intake by the mice was synthesized to glycogen in the liver for storage [38]. The generation of the hepatocyte vacuoles is also a normal physiological phenomenon observed in healthy liver, where the liver cells uptake the normal saline or transduced hUC-MSCs from the blood [39,40]. Overall, histology assessments of the major organs did not show any adverse events related to the treatment.

At the same time, there was no sign of immune rejection observed when allogenic transduced hUC-MSCs were infused into another species, the BALB/c mice. Indeed, previous studies have proved that hUC-MSCs possess low immunogenicity due to the low expression of class I and II major histocompatibility complex [7] and able to suppress the proliferation of activated lymphocytes in a xenograft model [8]. In particular, in the preclinical safety and toxicity evaluation of DSCs in BALB/c mice and Sprague–Dawley rats [23], and human BM-MSCs in Sprague–Dawley rats and New Zealand White (NZW) rabbits [41], has also proven that immune-competent animals treated with human source stem cells showed no immediate or long term side effects. In fact, our group also disclosed single and multiple xenotransplantation of hUC-MSCs through i.v. infusion had no short- and long-term toxicity in healthy BALB/c mice and no tumour formation in immunocompromised B-NDG mice [11].

Our findings concur with the results of other studies. It is, however, important to note that hUC-MSCs isolated from different umbilical cord sources, cultured and expanded using different protocols can contribute to MSCs heterogeneity. Consequently, cellular stability, its proliferation rate, secretions and other biological functions and properties will be affected to a greater or lesser extent [5,42]. Hence, it was crucial for us to evaluate the biodistribution and safety of our own hUC-MSCs, and to verify that they function similarly to others reported elsewhere. Collectively, we demonstrated that i.v. administration of hUC-MSCs did not cause any abnormality and toxicity in the major organs, and hUC-MSCs were eventually cleared from the body, confirming that hUC-MSC infusion is safe.

## 5 Conclusion

Human umbilical cord-derived mesenchymal stem cells (hUC-MSCs) can be successfully transduced with GFP-Luc2 lentivirus vector with stable GFP signal for up to 14 days *in vitro*. Systemic administration of hUC-MSCs at the dosage of 18.5 × 10^6^ cells/kg BW is safe and well tolerated in healthy BALB/c mice. Upon i.v. infusion, the transduced cells first localise predominantly in the lungs of healthy BALB/c mice and mainly remain in the lungs area for up to 3 days before eventually cleared from the body. In addition, the physical, biochemical and histological assessments show no toxicity or abnormalities throughout the study period. These results provide a solid foundation to our understanding of hUC-MSCs safety, homing and clearance in living hosts. Such information are essential and crucial in aiding the translation and development of safe and effective cellular therapies.

## Declaration of competing interest

SP Chin advices Cytopeutics on regulatory, clinical and research activities.

Information pertaining to writing assistance: No funded writing assistance was utilized in the production of this manuscript.
